# The antihelminth drug rafoxanide reverses chromosomal-mediated colistin-resistance in *Klebsiella pneumoniae*

**DOI:** 10.1128/msphere.00234-23

**Published:** 2023-09-25

**Authors:** Rongjia Han, Jiabao Xing, Huarun Sun, Zeyu Guo, Kaifang Yi, Gongzheng Hu, Yajun Zhai, Tony Velkov, Hua Wu

**Affiliations:** 1 Department of Pharmacology and Toxicology, College of Veterinary Medicine, Henan Agricultural University, Zhengzhou, China; 2 Key Laboratory of Zoonosis Prevention and Control of Guangdong Province, College of Veterinary Medicine, South China Agricultural University, Guangzhou, China; 3 College of Animal Science and Veterinary Medicine, Henan Institute of Science and Technology, Xinxiang, China; 4 National Reference Laboratory of Veterinary Drug Residues (SCAU), College of Veterinary Medicine, South China Agricultural University, Guangzhou, China; 5 Department of Pharmacology, Biodiscovery Institute, Monash University, Clayton, Victoria, Australia; Antimicrobial Development Specialists, LLC, Nyack, New York, USA

**Keywords:** rafoxanide, colistin resistance, chromosomal-mediated colistin-resistant *Klebsiella pneumoniae*, reversing resistance, transcriptomic analysis

## Abstract

The emergence and rapid spread of multi-drug-resistant (MDR) bacteria pose a serious threat to global healthcare. Although the synergistic effect of rafoxanide and colistin was reported, little is known regarding the potential mechanism of this synergy, particularly against chromosomal-mediated colistin-resistant *Klebsiella pneumoniae*. In the present study, we elucidated the synergistic effect of rafoxanide and colistin against chromosomal-mediated colistin-resistant *Klebsiella pneumoniae* isolates from human (KP-9) and swine (KP-1) infections. Treatment with 1 mg/L rafoxanide overtly reversed the MIC max to 512-fold. Time-kill assays indicated that rafoxanide acted synergistically with colistin against the growth of KP-1 and KP-9. Mechanistically, we unexpectedly found that the combination destroys the inner-membrane integrity, and ATP synthesis was also quenched, albeit, not via F_1_F_0_-ATPase; thereby also inhibiting the activity of efflux pumps. Excessive production of reactive oxygen species (ROS) was also an underlying factor contributing to the bacterial-killing effect of the combination. Transcriptomic analysis unraveled overt heterogeneous expression as treated with both administrations compared with monotherapy. Functional analysis of these differentially expressed genes (DEGs) targeted to the plasma membrane and ATP-binding corroborated phenotypic screening results. These novel findings highlight the synergistic mechanism of rafoxanide in combination with colistin which effectively eradicates chromosomal-mediated colistin-resistant *Klebsiella pneumoniae*.

**IMPORTANCE:**

The antimicrobial resistance of *Klebsiella pneumoniae* caused by the abuse of colistin has increased the difficulty of clinical treatment. A promising combination (i.e., rafoxanide+ colistin) has successfully rescued the antibacterial effect of colistin. However, we still failed to know the potential effect of this combination on chromosome-mediated *Klebsiella pneumoniae*. Through a series of *in vitro* experiments, as well as transcriptomic profiling, we confirmed that the MIC of colistin was reduced by rafoxanide by destroying the inner-membrane integrity, quenching ATP synthesis, inhibiting the activity of the efflux pump, and increasing the production of reactive oxygen species. In turn, the expression of relevant colistin resistance genes was down-regulated. Collectively, our study revealed rafoxanide as a promising colistin adjuvant against chromosome-mediated *Klebsiella pneumoniae*.

## INTRODUCTION

*Klebsiella pneumoniae* is a Gram-negative bacilli that often colonizes the oral cavity, intestinal tract, and skin of humans and animals, which can cause pneumonia, urinary tract infections, and sepsis (1, 2). The accelerating emergence and spread of multidrug resistance-resistant (MDR) *Klebsiella pneumoniae (K. pneumoniae*), including Carbapenem-resistant *Klebsiella pneumoniae* (CRKP) in our hospitals and the community, promoted a resurgence in the use of polymyxin B and colistin. These important last-line lipopeptide antibiotics were reconsidered into our administration option due to their unique mechanism, albeit they were banned in hospitals in the 1970s due to side effects such as nephrotoxicity and neurotoxicity (3). Without novel antibiotics in the near future, polymyxins are increasingly used as the last-line therapy against this dangerous pathogen. In November 2015, polymyxin resistance mediated by a plasmid-borne phosphoethanolamine (pEtN) transferase gene, *mcr-1*, was reported and generated significant concerns of potential rapid dissemination of polymyxin resistance via horizontal transfer (4). Since then, eight *mcr* genes (*mcr*-1 to *mcr*-9 except *mcr*-6) have been discovered in several Gram-negative species, including *K. pneumoniae. K. pneumoniae* is a serious nosocomial pathogen and can rapidly evolve to gain MDR status, limiting the treatment options to polymyxin B or colistin. Polymyxins target the Gram-negative outer membrane and resistance is mainly due to modifications of the lipid A component. The most common mechanism of polymyxin resistance in *K. pneumoniae* is modifications of the lipid A phosphates with positively charged moieties [e.g., 4-amino-4-deoxy-l-arabinose (l-Ara4N), pEtN, and/or galactosamine] that repel the cationic polymyxin molecule. Lipid A modifications with pEtN can be mediated by either a chromosomally encoded pEtN transferase gene, *pmrC*, or a plasmid-encoded *mcr* gene. In contrast, l-Ara4N modification of lipid A is mediated by a transferase encoded by *arnT* (pmrK) that is located exclusively on the chromosome (5, 6). Synergistic combinations of individually ineffective drugs confer a potential solution to the costly *de novo* development of novel antibiotics. In this respect, the concept of “repurposing” (the search for new uses for existing drugs) has gained considerable interest (7).

Rafoxanide is an antihelminth veterinary drug belonging to the salicylanilide family that is commonly used against fluke infections in ruminants. Two preliminary reports highlighted the synergistic effect of the combination of rafoxanide and colistin against *K. pneumoniae* (8, 9). In the present study, we employed a meta-transcriptomic approach in an effort to understand the underlying synergistic mechanism of rafoxanide in combination with colistin, as well as the molecular mechanism(s) of resistance rescue against the non-plasmid-mediated colistin-resistant *K. pneumoniae* strains isolated form human and swine sources.

## MATERIALS AND METHODS

### Strains and reagents

The bacterial strains KP1 and KP9 were isolated from piggery wastewater and patient who is hospitalized in The Third Affiliated Hospital of Henan University of Chinese Medicine, respectively. Strains were cultured in Luria-Bertani (LB) broth or MacConkey medium at 37°C overnight. Taxonomic classifications were confirmed by 16S rRNA using Sanger Sequencing. All chemicals used in this study were purchased from Zhengzhou Boxiang Biotechnology Co., Ltd and Henan Tianchi Biotechnology Co., Ltd.

### Antimicrobial susceptibility assay

The antibacterial activity of colistin *in vitro* was assessed by microbroth dilution susceptibility testing according to the current CLSI guidelines (10). In a 96-well plate containing cation-adjusted MHB broth, a twofold dilution drug was prepared, and then an equal volume of bacterial suspension was added and then incubated at 37°C for 18 h. MIC values for colistin were determined as the lowest concentration to inhibit visible bacterial growth in the form of turbidity. According to current break-points, we considered MIC ≥ 4 mg/L as colistin-resistant. Wells with or without bacterial cells were used as positive or negative controls, respectively.

### Whole-genome detection

*Mcr* family genes (*mcr* 1–5 and 7–9) were amplified by polymerase chain reaction (PCR), and we found that no *mcr*-family gene was detected. Then to find the resistant genes at the chromosome, the isolates were whole-genome sequenced using an Illumina Nextera XT Library Prep Kit and Illumina MiSeq sequencing platform (Illumina Inc., San Diego, CA) with 300 bp paired-end reads. Annotation of the genomes was performed with Rapid Annotation using Subsystem Technology (RAST) (http://rast.nmpdr.org/) and PATRIC (https://www.patricbrc.org/). ResFinder, PlasmidFinder, VirulenceFinder, and Multi-Locus Sequence Typing (MLST) tools (https://cge.cbs.dtu.dk/) were used for further analysis. Shortly, the mutations of the resistant genes were determined by aligning with the reference *crrA/B*, *pmrA/B*, *phoP/Q*, and *mgrB* genes of *K. pneumoniae* ATCC 35657.

### Checkerboard assay

We deployed a checkerboard assay to determine the synergistic activity of the rafoxanide and in combination with colistin. Briefly, colistin and rafoxanide were first 2-fold serially diluted 13 times along *x*-axis analogous to the monotherapy assay, respectively. Then, both diluted agents were mixed evenly corresponding to horizontal and longitudinal coordinates in the 96-well plate, left with one row for monotherapy control. Overnight cultured bacteria were then diluted in saline to 0.5 McFarland turbidity, with 1:100 dilution in MHB broth for inoculation on each well. Similarly, wells with or without bacterial cells were used as positive or negative controls, respectively. The assays were interpreted using the fractional inhibitory concentration (FIC) index that was calculated by dividing the MIC of colistin in combination with rafoxanide by the MIC of colistin alone. The FIC of rafoxanide was calculated in line with colistin. The FIC index (FICI) was the summation of both FIC value. The formula is as follows (11).


$$FICI\;=\;FICI_{a}\;+\;FICI_{b}\;=\;MIC_{ab}\;/\;MIC_{a}\;+\;MIC_{ba}\;/\;MIC_{b}$$

### Time-kill kinetics assays

Overnight cultured bacteria were diluted using LB broth in saline to 0.5 McFarland turbidity, followed by 1:100 dilution using LB broth (final volume 10 mL). Diluted bacteria were then incubated with colistin, rafoxanide, and combination administration, respectively. One hundred microliters of aliquot was acquired from each group at 0, 2, 4, 8, 12, and 24 h, respectively, centrifuged, and resuspended in PBS. The bacterial colonies were then counted by overnight incubation on the LB agar plates at 37°C.

### Fluorescence assays

Pretreatments of biochemical assays were performed using similar protocols as previously described in detail. Specifically, KP1 and KP9 were grown overnight at 37°C on the shaker at 200 rpm/min. Then, the cultures were washed and suspended with 5 mmol L^−1^ HEPES (pH 7.0, plus 5 mmol L^−1^ glucose). In the same buffer, the OD600 of the bacteria suspension was standardized to 0.5 and the fluorescent dye was added. After incubation at 37°C for 30 min, an aliquot of 1 mL of bacterial suspension was mixed with the final concentration of rafoxanide or colistin alone or in combination. After incubation for 1 h, 200 µL bacterial suspension was added to the 96-well plate. Subsequently, fluorescence intensity or luminescence was measured by a Spapk 10 M Microplate reader (Tecan) (12).

### Outer membrane permeability assay

The fluorescent probe 1-*N*-phenylnaphthylamine (NPN) was employed to measure changes in the outer membrane (OM) permeability of KP1 and KP9 in response to antibiotic treatment. Fluorescence intensity was measured with the excitation wavelength at 350 nm and emission wavelength at 420 nm (13).

### Cell membrane integrity assay

The fluorescence intensity of propidium iodide (PI)-labeled (Beyotime, Shanghai, China) cells in the presence of increasing drugs was measured with the excitation wavelength of 535 nm and emission wavelength of 615 nm.

### Proton motive force assay

3,3′-Diethyloxacarbocyanine iodide [DiOC2(3)] (30 µM) (Yuanye, Shanghai, China) was used to determine the membrane potential (Δ*ψ*). Dissipated membrane potential of KP1 and KP9 were measured with the excitation wavelength of 486 nm and emission wavelength of 620 nm (14). The pH gradient (ΔpH) was detected by deploying the pH-sensitive fluorescent probe BCECF-AM (20 µM) (Beyotime, Shanghai, China). For all BCECF experiments, the excitation and emission wavelengths of the fluorescence spectrometer were set as 500 and 522 nm, respectively (12).

### Reactive oxygen species detection

We employed 2′,7′-dichlorodihydrofluorescein diacetate (DCFH-DA, 10 µM) to detect the level of KP1 and KP9. The excitation wavelength and emission wavelength were 488 and 525 nm to measure the fluorescence intensity, respectively (15).

### Intracellular ATP

Intracellular ATP levels of KP1 and KP9 were identified using an enhanced ATP assay kit (Beyotime, Shanghai, China). Overnight cultured strains were washed with 0.01 mg/L PBS (pH 7.4) two times and then resuspended to obtain an OD600 of 0.5. After treating with different concentrations of colistin alone or in combination with rafoxanide for 1 h, bacterial cultures were centrifuged and the supernatant was removed. Bacterial precipitates were lysed by lysozyme, centrifuged, and the supernatant was used for intracellular ATP levels measurement.

### EtBr efflux assay

The prepared suspension was washed three times with 10 mM HEPES buffer and dispensed the OD600 = 0.3, followed by the addition of 5 µM EtBr incubated 30 min at 37°C. An aliquot of 1 mL of bacterial suspension containing the indicated concentration of rafoxanide or colistin alone or in combination or 100 µM CCCP *per se*. Incubation for 1 h, then the fluorescence was monitored at excitation/emission of 525/600 nm at 37°C with a luminometer (Varioskan Flash; Thermo Scientific) (16).

### Scanning electron microscope

To visually verify the damage of cell membrane, we applied a scanning electron microscope (SEM) to observe the bacterial colonies morphology with different treatments. Hence, we refined the experiment protocol to observe the bacterial morphology. Specifically, the overnight cultured bacterial colonies were incubated into LB broth with 1:100 (final volume 10 mL), and then the LB broth was amplified on the shaker at 37°C for 6 h. Subsequently, the agents with different treatment strategies were incubated and further cultured on the shaker at 37°C for 18 h. Finally, the samples were fixed with 2.5% glutaraldehyde at 4°C for 24 h. Then, the bacteria were gradually dehydrated with ethanol (30%, 50%, 70%, 90%, and 100%). The processed samples were dried using a critical point dryer and coated with a layer of gold-palladium using an ion sprayer and observed with SEM (Environmental Scanning Electron Microscope Q45). Final images were implemented by Wuhan Saiville Biotechnology Co., Ltd.

### Transcriptomic analysis

To better understand the effect of rafoxanide and colistin on *K. pneumoniae* on gene expression, we performed transcriptomic sequencing of *K. pneumoniae* in response to treatments. The treatment conditions were as follows: KP1 was exposed to 64 mg/L colistin and 1 mg/L rafoxanide separately, in combination, and blank control. Subsequently, overnight cultured bacterial colonies were incubated into LB broth with 1:100 (final volume 10 mL), and then, the bacteria colonies were further amplified on the shaker at 37°C for 6 h. The agents with different treatment strategies were incubated into the suspensions, respectively, and the suspensions were further cultured on the shaker at 37°C for 4 h. An untreated control tube was prepared in parallel with each experiment. Then, the bacterial RNA was isolated using TruSeqTM Stranded Total RNA Library Prep Kit for Illumina sequencing. Samples were sequenced on the Illumina HiSeq platform with PE150 paired reads. The quality of the resulting fastq reads we checked using FastQC v0.11.9 (Babraham Bioinformatics, Cambridge) and mapped on the reference genome using Bowtie2 v2.4.2 using default settings. The resulting SAM files were converted to BAM using SAMtools 1.11, and Feature Counts 2.0.1 was used to get the gene counts. The T-REx webserver was used to perform statistical analysis and determine differential gene expression (DGE), and subsequently, gene set enrichment analysis was performed for functional analysis using the GSEA-Pro web server (http://gseapro.molgenrug.nl).

### Statistical analysis

Statistical analysis was carried out using GraphPad Prism 8.0.2 and SPSS v26. All data were presented as mean ± standard deviation. The unpaired *t*-test between the two groups (normally distributed data) or one-way ANOVA between the groups (one-way ANOVA) was employed to calculate the *P*-value (*P < 0.05, **P < 0.01, and ***P < 0.001).

## RESULTS

### *In-vitro* synergistic effect of rafoxanide and colistin against wild-type *K. pneumoniae*

**Fig 1:**
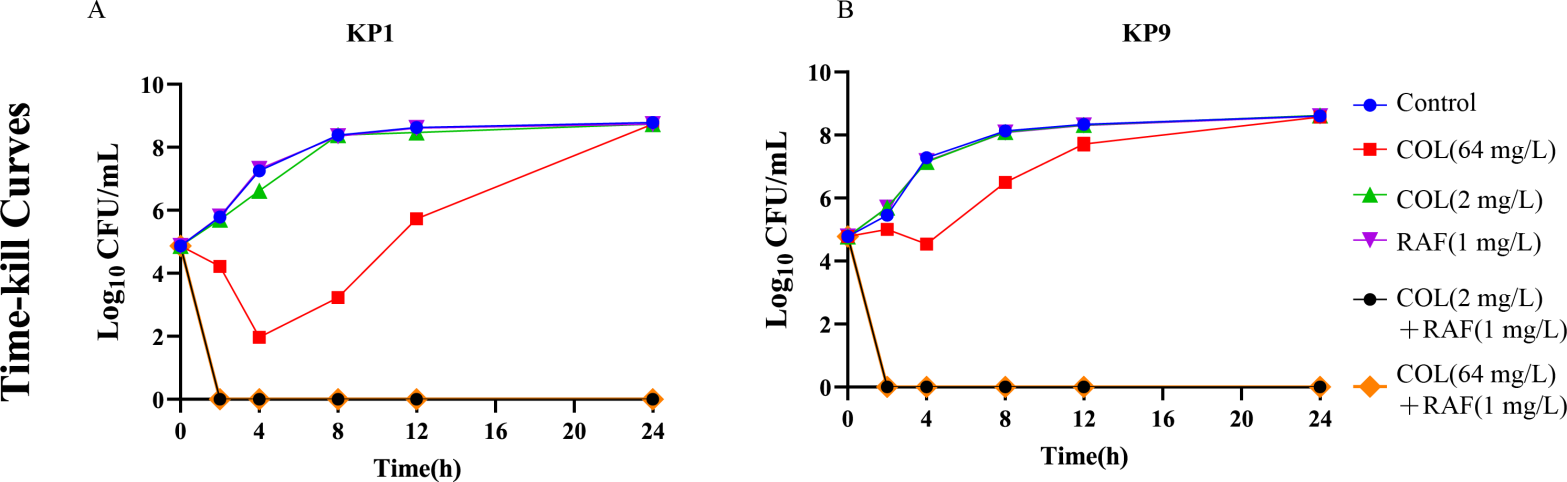
Time-kill curves of KP1 and KP9 after treatment of rafoxanide and colistin. KP1 was grown to exponential growth phase and then treated with colistin (1/2 MIC colistin and 1/64 MIC colistin) or rafoxanide (1 mg/L) alone or in combination for 24 h. Data are representative of three independent experiments and shown as mean ± SD.

We first detected the antibacterial effect of rafoxanide (RAF) and colistin alone against the novel isolated human-source *K. pneumoniae* strain KP-9 and swine-source *K. pneumoniae* strain KP-1. As shown in Table 1, rafoxanide and colistin administration alone showed a weak antibacterial effect, respectively (i.e., MIC >512 mg/L and MIC = 128 mg/L) (Table 1). While, rafoxanide and colistin in combination overtly reversed the antibacterial effect of colistin to the minimum MIC of 0.25 mg/L. Besides, as we adjusted the concentration of rafoxanide to combine with colistin to test the antibacterial effect manifested by MIC, the synergy effect, i.e., MIC value, would not be affected by the different concentrations of rafoxanide. Hence, we determined to deploy 1 mg/L of rafoxanide in the following assays. To further corroborate the potential interaction relationship between rafoxanide and colistin, we carried out the checkerboard assay, which showed synergistic interaction against both isolates (Table 1). Time-kill assay indicated that 2 mg/L colistin alone could not inhibit bacterial growth, and 1/2 MIC (64 mg/L) colistin inhibited the growth of both isolates during 0–4 h, whereas thereafter, the bacterial concentrations were recovered (Fig. 1). On the contrary, as we combined the RAF with 2 mg/L or 64 mg/L colistin, both of KP-1 and KP-9 strains were significantly antagonized after 24-h exposure.

**TABLE 1:** MIC of the strains^*a*^

Strain	Source	MIC_RAF_	MIC_COL_	MIC_COL_[combo]						FIC index	Interpretation
				0.25 mg/L RAF	0.5 mg/L RAF	1 mg/L RAF	2 mg/L RAF	4 mg/L RAF	8 mg/L RAF		
KP1	Swine	＞512	128	1	1	0.25	0.25	0.25	0.25	0.0078 ＜ *X* ＜ 0.0088	Synergy
KP9	Human	＞512	128	16	4	1	1	1	1	0.0078 ＜ *X* ＜ 0.0088	Synergy

^*a*^ COL, colistin; RAF, rafoxanide.

PCR detection found that there is no *mcr* gene detected in both strains. Then, we tried to find the potential resistance genes at the chromosome. In KP9, Pro135Ser was detected in *crrA* and Ile27Val was detected in *crrB*. In *mgrB*, two different insertion sequence (IS) elements were detected, respectively. In KP1, we detected a 777-bp insertion (IS1-like) started at 123 nt, and a 1,066-bp insertion (IS903B-like) was identified in KP9. Detailed information was listed in Table 2.

**TABLE 2:** Genetic information of the strains^*a*^

Strain	mcr family	Gene locus status						
		CrrA	CrrB	PrmA	PrmB	PhoP	PhoQ	MgrB
KP1	–	–	–	–	–	–	–	Insertional inactivation (777 bp) by IS1-like at nt 123
KP9	–	Pro135Ser	Ile27Val	–	–	–	–	Insertional inactivation (1,066 bp) by IS903B-like at nt 66

^*a*^ mcr family, mcr1–5, 7–9; IS, insertion sequence. - means this mutation was undetected.

### Combined administration of rafoxanide and colistin synergistically damages the cytoplasmic membrane of *Klebsiella pneumoniae* whereas rarely permeate the outer membrane

**Fig 2:**
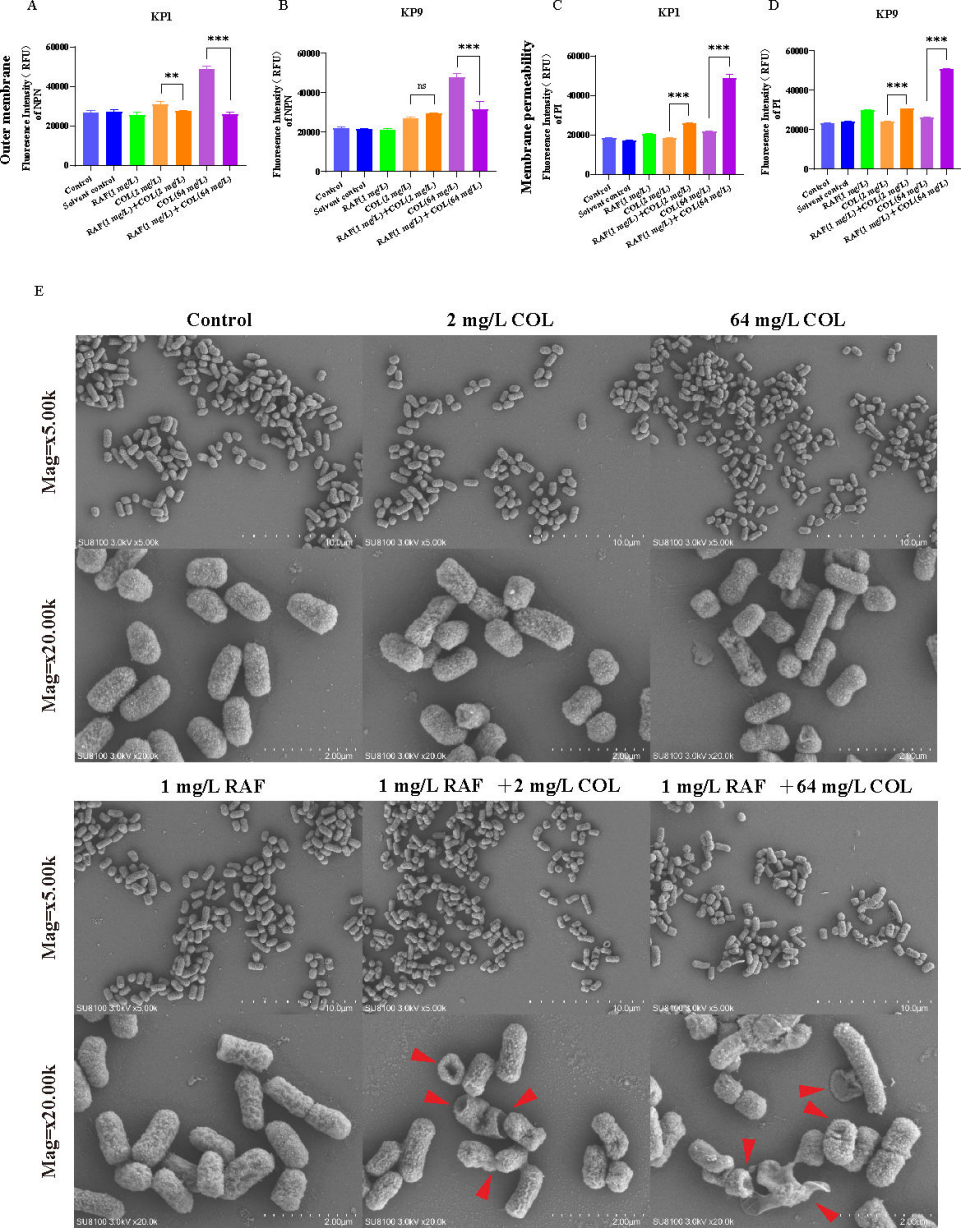
The effect of rafoxanide and colistin against cell membrane permeability. (**A and B**) Rafoxanide did not enhance the outer membrane disruption of colistin (1/2 MIC colistin and 1/64 MIC colistin) in KP1 (**A**) and KP9 (**B**). (**C and D**) Rafoxanide significantly enhances the inner membrane permeability in KP1 (**C**) and KP9 (**D**). Permeability was evaluated by measuring the fluorescence intensity of propidium iodide after exposure to increasing concentrations of colistin (1/2 MIC colistin and 1/64 MIC colistin), constant rafoxanide, or colistin plus rafoxanide for 1 h. Unpaired *t*-test between two groups or one-way ANOVA among multiple groups were used to calculate *P*-values (***P < 0.001). (**E**). Morphological observation KP9 after different treatments (Top panel: 5,000×, bottom panel: 20,000×).

As exemplified by previous studies, rafoxanide could strengthen the bactericidal effect of colistin against colistin-susceptible but more importantly colistin-resistant Gram-negative bacilli, which, however, did not fully explain the observed synergism. Therefore, we first measured the outer membrane permeability of *K. pneumoniae* under different concentrations of rafoxanide plus colistin treatment using the hydrophobic fluorescent probe 1-*N*-phenyl naphthyl amine. NPN is a hydrophobic fluorescent probe that releases fluorescence when interacting with the hydrophobic parts of the phospholipid bilayer, which was used to detect the permeability of the outer membrane (17). In this study, we found that the fluorescence intensity of two agents in combination was unexpectedly reduced compared with the monotherapy of colistin both in different concentrations, which suggested that the outer membrane may not be damaged by colistin adhesion (Fig. 2A and B). To test the effect of rafoxanide regarding the outer membrane permeability, we further deployed the distinct gradient of rafoxanide concentration, i.e., 1, 2, and 4 mg/L in combination with 64 mg/L colistin, which suggested that fluorescence intensity of NPN was negatively correlated with the concentration gradient of rafoxanide (Fig. S1). Subsequently, we evaluated the effect of combined treatment on bacterial cytoplasmic membrane (i.e., inner membrane) integrity using the DNA-binding dye propidium iodide, which showed that the combined administration of colistin and rafoxanide could amplify the fluorescence intensity compared with colistin alone, suggestive of that rafoxanide and colistin could synergistically destruct the cytoplasmic membrane in a dose-response manner (Fig. 2C and D).

To verify the biological change on a morphological basis, we further observed the morphology of the cytoplasmic membrane on the KP-9 strains after different administration processing using scanning electron microscopy (SEM). We analogously did not observe overt damage of cytoskeleton membrane as we treated with monotherapy, i.e., 2 mg/L, 64 mg/L colistin, and 1 mg/L rafoxanide, while in both groups of combined treatment, the membrane was disrupted to a varying degree without exception (Fig. 2E). Specifically, we did not observe significant membrane disruption in the control group, 2 mg/L colistin group, 64 mg/L colistin group, and 1 mg/L rafoxanide group. As treated with 2 mg/L of colistin and rafoxanide in combination, the bacterial envelope was partially disrupted, and leakage of the cytosol could be observed, along with shrinkage of the cell membrane. Treatment with 64 mg/L colistin and rafoxanide resulted in more severe disruption of the inner membrane, and more cytoplasmic exudation on the membrane surface. These observations suggested that the presence of rafoxanide could salvage the killing effect of colistin on CoRKP in other manners rather than destructing the outer membrane-associated cytoskeleton (Fig. 2E), which piqued our interests into other biological effects to *K. pneumoniae*.

### Synergistic administration of rafoxanide and colistin damages the ATP synthesis, not through proton motive force dissipation of *Klebsiella pneumoniae*

**Fig 3:**
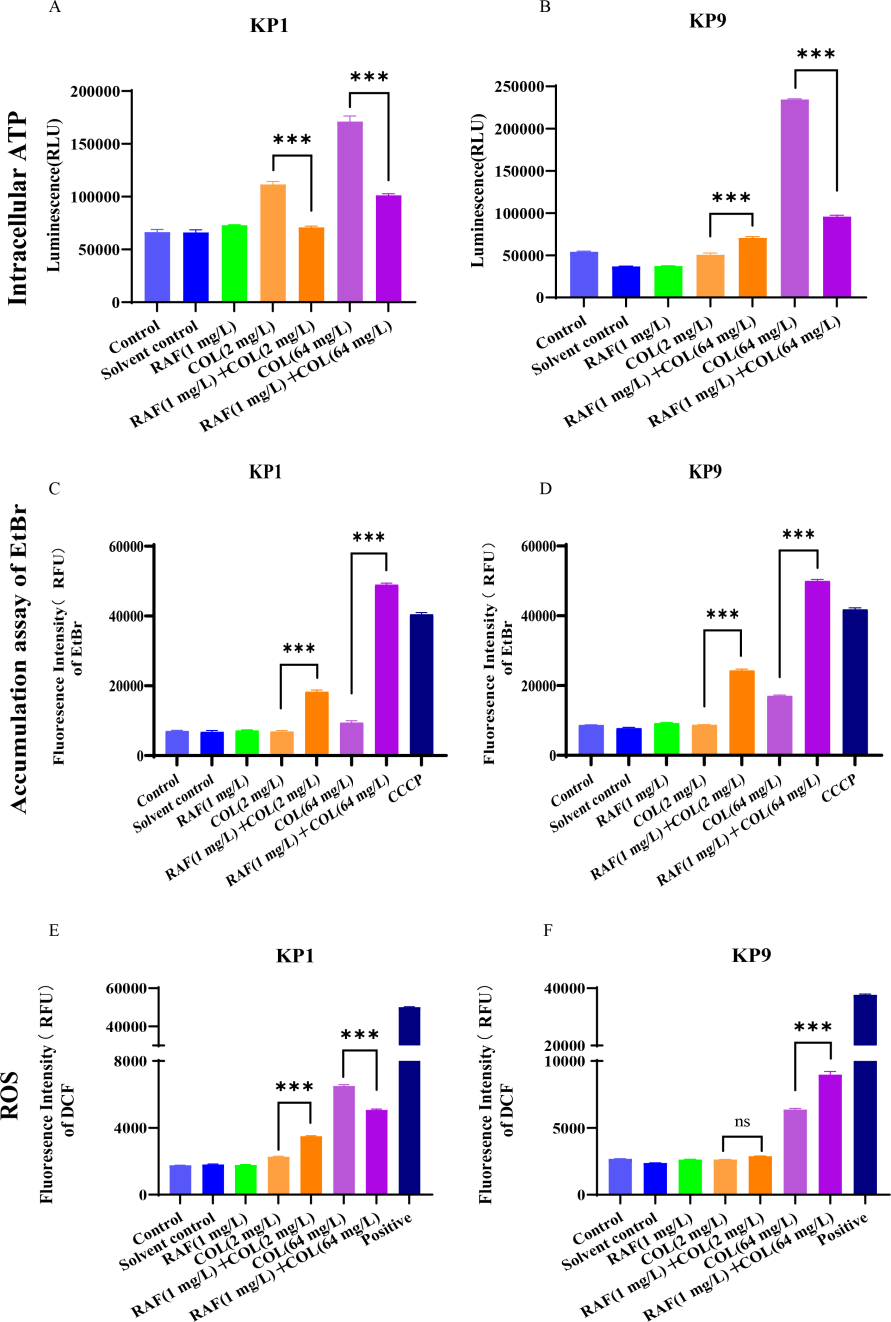
The ATP production, EtBr efflux inhibition, and ROS detection treated with rafoxanide and colistin against KP1 and KP9. (**A and B**). The ATP production changes when KP1 (**A**) and KP9 (**B**) were treated with rafoxanide and colistin (1/2 MIC colistin and 1/64 MIC colistin), respectively, or combined administration. (**C and D**). EtBr efflux inhibition treated with rafoxanide and colistin against KP1 and KP9. Efflux pump inhibition detection when KP1 (**C**) and KP9 (**D**) were treated with rafoxanide and colistin (1/2 MIC colistin and 1/64 MIC colistin), respectively, or combined administration. CCCP was deployed as the positive control. (**E and F**). Excessive ROS generation treated with rafoxanide and colistin against KP1 and KP9. Excessive ROS generation when KP1 (**E**) and KP9 (**F**) were treated with rafoxanide and colistin (1/2 MIC colistin and 1/64 MIC colistin), respectively, or combined administration. Rousp was deployed as a positive control. Unpaired *t*-test between two groups or one-way ANOVA among multiple groups were used to calculate *P*-values (***P < 0.001).

The increase in membrane permeability usually causes the dissipation of the membrane potential (18). Due to our abnormal observations regarding the membrane change, hence, we further hypothesized that the membrane potential would rather than be destructed, i.e., depolarization. Hence, we further investigated the membrane potential of the cells treated with rafoxanide plus colistin using the potential-sensitive membrane dye DiOC2(3). It indicated that the combined administration of different concentrations significantly reduced the fluorescence activity, to different degrees, respectively, suggestive of the unaffected potential difference (Fig. S2). Because membrane potential and the transmembrane pH gradient (the difference between the intracellular and extracellular pH) are interdependent (19), unaffected membrane potential would, therefore, be presumed a steadiness pH value. Hence, we detected the ΔpH against different dosage regimens, which corroborated our presumption that the ΔpH is no significant change (Fig. S3). These observations suggested that the PMF is not significantly dissipated by the combined administration. Considering that the PMF is subsequently necessary for ATP synthesis by the F_1_F_0_-ATPase and for the transport of various solutes, we wondered if the ATP would vary when rafoxanide and colistin are co-treated. We measured the level of ATP and found that, compared with the mono-administration of colistin, combination administration significantly inhibited the synthesis of ATP (Fig. 3A and B). Collectively, these results suggested that combined administration of rafoxanide and colistin quenched the growth of *K. pneumoniae* partially by inhibiting the synthesis of ATP, where the ATP synthesis inhibition is not through PMF dissipation.

### Synergistic suppression of RAF and colistin against efflux pump of *Klebsiella pneumoniae*

Sufficient accumulation of antibiotics in cells is a prerequisite for antibacterial activity, especially for Gram-negative pathogens. We further hypothesized that the ATP synthesis reduction caused by the combined administration of RAF plus colistin might have an impact on the function of the efflux pump on cell membrane. Hence, we deployed the efflux pump accumulation assay to verify the specific function. We observed a drastic accumulation of the substrate EtBr in the combinatorial administration of RAF and colistin compared with the separate administration of both. To be specific, a more significant increase in the fluorescence intensity of EtBr was observed after we processed the colistin from 2 mg/L to 64 mg/L, indicative of that the effect of the inhibition of colistin on the efflux pump was acting in a dose-dependent way. The inhibition level of combination administration is evenly higher than the Cyanochlorophenylhydrazone (CCCP) disposed group (Fig. 3C and D). These results suggested that in the presence of rafoxanide, the effect of efflux pump in *K. pneumoniae* would be significantly minified, thereby accumulating intracellular drugs.

### Synergistic administration of RAF and colistin compromise oxidation reaction of *Klebsiella pneumoniae*

The excess concentration of intracellular reactive oxygen species (ROS) may partly lead to uncontrollable damage of bacteria. As of KP-1, we did not observe significant fluorescence activity upgrade after administration alone of 2 mg/L colistin and RAF, respectively. In contrast, RAF in combination with 2 mg/L colistin significantly enhanced the fluorescence activity compared with the colistin-processing group. However, albeit we observed the ascending trend of fluorescence activity in both of 64 mg/L colistin group and combination group of the 64 mg/L colistin and RAF, RAF in the combination of 64 mg/L colistin significantly decreased the fluorescence activity, compared with 64 mg/L colistin single administration, where the trend does not coincide with the group of lower concentration of colistin (i.e., 2 mg/L colistin). As of KP-9, we did not observe a significant enhancement of fluorescence activity in the administration of RAF in combination with 2 mg/L colistin compared with a single administration of 2 mg/L colistin. On the contrary, the combination administration group of RAF and 64 mg/L colistin significantly increased the fluorescence activity, compared with 64 mg/L colistin single administration (Fig. 3E and F). Collectively, these results jointly indicated the treatment of RAF enhanced the bactericidal effect of colistin attributable to increase oxidative stress.

### Transcriptomic response of *Klebsiella pneumoniae* to rafoxanide and colistin combined administration

**Fig 4:**
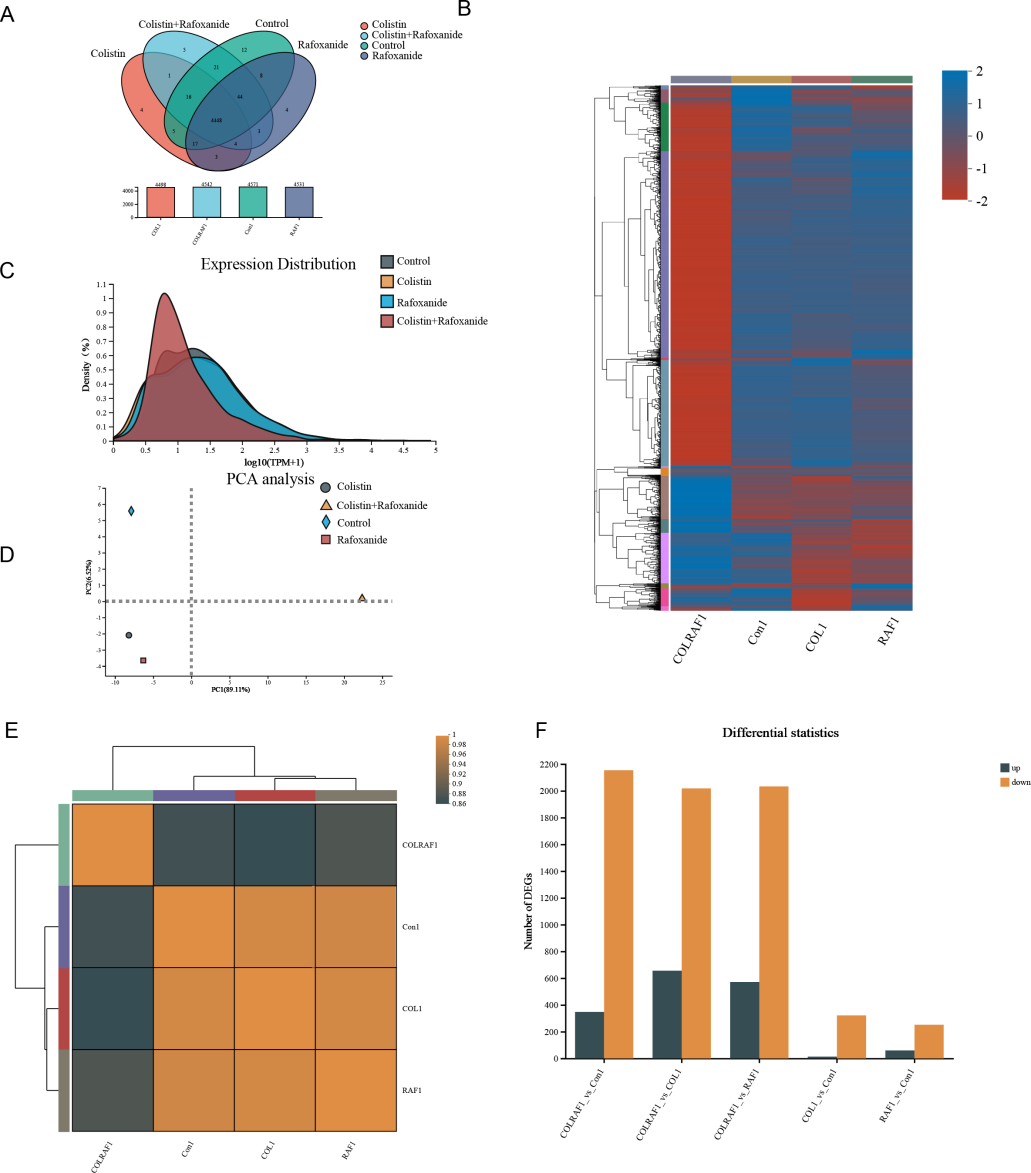
Transcriptome profile of KP1 unraveled a heterogeneous gene expression landscape. (**A**) Venn diagram showing the gene expression difference with different treatments against KP1, with the bottom panel showing the detected total gene numbers. (**B**) Heatmap plots showing the proportion of differentially expressed genes after different administration treatments against KP1, which showed significant down-regulating profiling. (**C**) The TPM (transcripts per kilobase of exon model per million mapped reads) comparison indicated a heterogeneous expression landscape with different administration treatments. (**D**) Principal Component Analysis (PCA) analysis of the gene expression profiles with different administration treatments. (**E**) Correlation analysis of gene expression profiles with different administration treatments. (**F**) Differential statistic of differential-expressed genes with different administration treatments. COL1, 64 mg/L colistin treated KP1 group; RAF, 1 mg/L rafoxanide treated KP1 group; and Con1, KP1 without any treatment.

In total, 4,448 concurrent genes were detected against different treatment groups, with 4, 4, 5, and 12 solely screened in colistin, rafoxanide, colistin plus rafoxanide group, and untreated group (Fig. 4A). The comparison of treatment with the combination of 1 mg/L rafoxanide plus 64 mg/L colistin to untreated cells revealed 2,153 downregulation of genes, while 347 genes were upregulated, and 570 genes upregulated when treated with 1 mg/L rafoxanide alone and 2,032 genes downregulated, and 655 genes upregulated with 2,017 genes downregulated compared with colistin treatment alone, whereas the monotherapy is not significantly affected the different mRNA expression (Fig. 4B, C and F). Principal-Component Analysis (PCA) of unsupervised learning suggested that the transcriptional responses of 1 mg/L rafoxanide and 64 mg/L colistin monotherapy were clustered tightly (Fig. 4D and E). In contrast, the untreated group and the combined administration group were clustered solely. These observations suggested a heterogeneous mRNA expression landscape. Hence, we further deployed Gene Ontology (GO) analysis to focus on the functional difference between different treatment strategies (Fig. S4). The integral component of membrane, plasma membrane, and cytoplasm was the most differential cellular component, whereas the outer membrane-associated difference of expression is not evidently mapped, which suggested that the cytoplasmic membrane is significantly affected in our combined administration group, further corroborating our results of heterogeneity against outer-inner membrane. The most differential expressed component we mapped in the molecular function is ATP binding.

**Fig 5:**
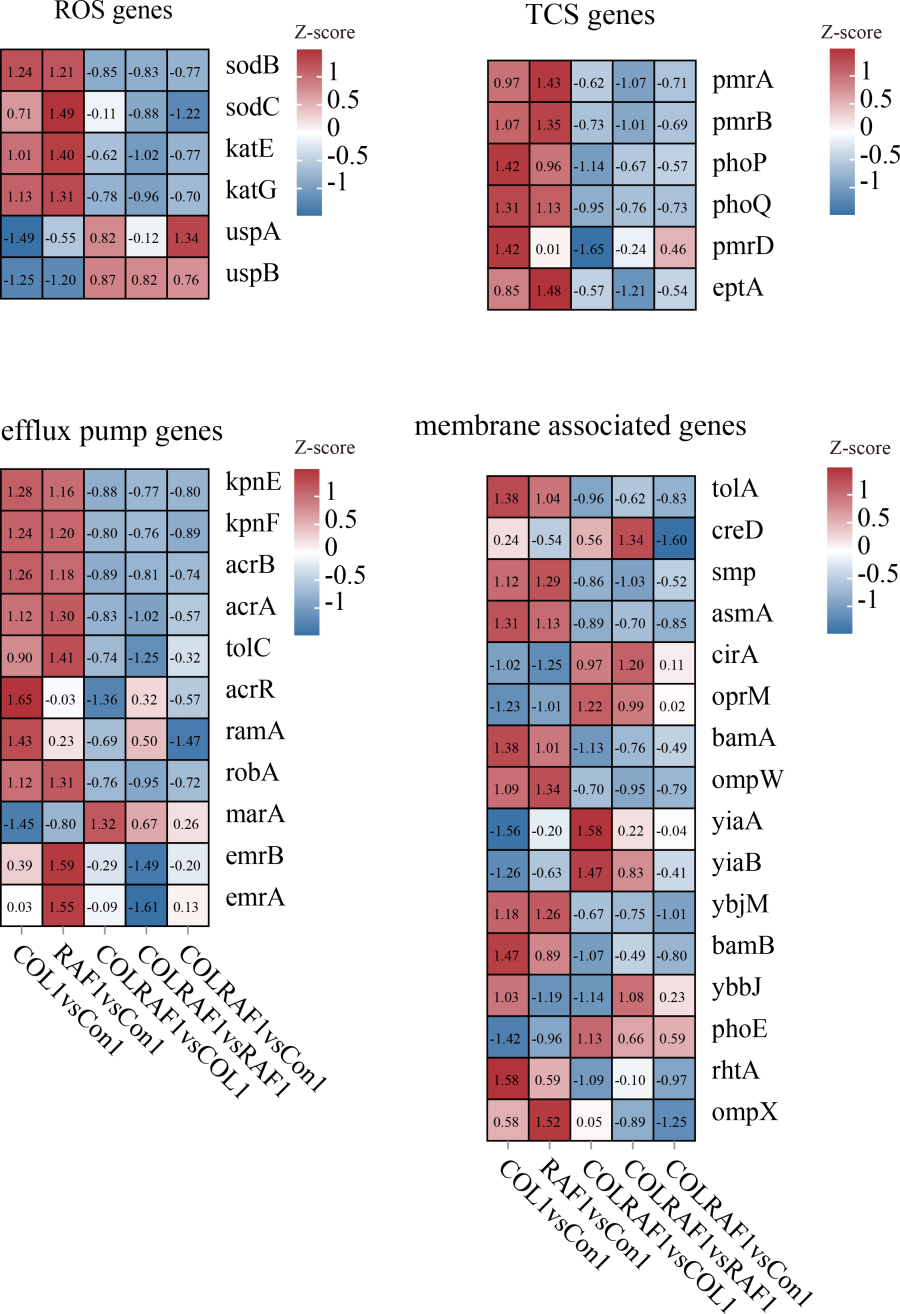
Functional analysis of transcriptomic profile. Selected DEGs involved in the antioxidant response, two-component system, multidrug efflux pump, and membrane regulation. COLRAF1: 64 mg/L colistin and 1 mg/L rafoxanide treated KP1 group, COL1: 64 mg/L colistin treated KP1 group, RAF: 1 mg/L rafoxanide treated KP1 group, and Con1: KP1 without any treatment.

Like most CoRKP strains, KP-1 carried chromosomal mutations rather than plasmid-mediated *mcr* families (20). Likely explaining its colistin resistance, the expression of *mgrB* gene is lacking in our transcriptomic gene screen, a negative regulator of the *PhoP/Q* two-component system, which directs resistance-conferring modification of colistin’s target on lipopolysaccharide molecules (Fig. 5). When exposed to the combined administration, the two-component systems such as *pmr A/B*, *phoP/Q*, as well as *pmrC* were significantly downregulated compared with the monotherapy and control groups (21) (Fig. 5). The damage to the oxidative phosphorylation process is involved in the intracellular accumulation of ROS. Treatment with rafoxanide plus colistin significantly reduced the expression of major oxidative stress-relief enzymes. In addition, among Multi-Drug-Resistance (MDR) determinants, Resistance-Nodulation-cell Division (RND) efflux pumps have been reported as the main contributors, due to their ability to extrude a wide variety of molecules out of the bacterial cell. *Tol C* and *acr A/B*, the efflux pumps belonging to the RND family, are energy-dependent, which are differentially expressed under combined administration, suggesting that the effective intracellular accumulation of drug molecules is significantly potentiated as rafoxanide entered into cells (Fig. 5). Interestingly, these findings suggested that the insertion of *mgrB* in both strains inactivated the *mgrB* gene in both isolates.

Together, these results demonstrated that rafoxanide specifically rescued colistin bactericidal activity via the increased accumulation of intracellular antibiotics, inner membrane damage, ATP synthesis, as well as excessive oxidative stress. As a result, the efflux pump function is inhibited, and intracellular ROS accumulates to enhance the efficacy of killing the bacteria.

## DISCUSSION

Colistin was considered one of the last-resort antibiotics on account of the emergence of MDR GNB. However, in most recent years, the horizontal widespread of *mcr* family pressed us to find an alternative way as a therapeutic strategy against colistin resistance (22, 23). As an anti-helminth drug, rafoxanide has been reported to inhibit the development of cancer cells (24 – 26), which does not have the ability to resist Gram-negative bacteria when chosen as monotherapy. As reported in Miró-Canturri et al. and Domalaon et al. studies, it showed a good synergistic effect against Gram-negative bacteria when combined with colistin, whereas its potential synergistic mechanism is still unclear yet.

In this study, we first examined the synergistic effect of both administrations against clinical CoRKP isolates. The MIC of colistin plus 1 mg/L rafoxanide against the clinical CoRKP isolates was decreased to approximately 512-fold, and both of time-kill assay and checkerboard assay further suggested a synergy effect, which is consistent with the results of Domalaon et al.’s study (9). And most CoRCP results from the invalidation of *mgrB*, thus involving the constitutive activation of the two-component system *PhoP/Q* and an increase in lipid A modifications and subsequent colistin resistance (27). Here, the resistance of both isolates results from the lacking of *mgrB* gene rather than plasmid mediation, i.e., *mcr* families.

Colistin plus rafoxanide significantly disrupted the cytoplasmic membrane manifested by the propropium bromide fluorescence intensity. However, as the *K. pneumoniae* was treated with colistin and rafoxanide in combination, the NPN fluorescence signal intensity rather than descended compared with colistin monotherapy for each concentration, suggesting that the outer membrane was not significantly disrupted as we expected, consistent with Kaur A (28) and Shruti Kashyap’s (29) findings. We hypothesized that, due to the positive charge properties of rafoxanide *in vivo*, the positive rafoxanide ions would competitively bind to colistin ions, invalidating the function of colistin target to the LPS of gram-negative bacterial outer membrane cleavage. Then as we deployed the concentration gradient of rafoxanide as 1, 2, and 4 mg/L in combination with colistin, the fluorescence intensity of NPN was strongly correlated with the concentration gradient of rafoxanide, corroborating our hypothesis.

The generation of ROS is regarded as one of the important aspects of antibiotics that induce oxidative stress in bacteria with the subsequent contribution to its lethality (30, 31). Of the ROS-generating pathways involved in bacterial lethality, causing a disturbance in metabolism and respiration. ROS relative level following treatment with colistin and rafoxanide in combination was potentiated. Hence, we further investigated the ROS-related gene expression difference with the treatment of rafoxanide and colistin, respectively, and combined administration. The expression of Fe-Mn superoxide dismutase (*sodB*) and monofunctional catalase (*katE*) showed a significantly decreased following treatment with the combination of colistin and rafoxanide. This reduced expression, in addition to the down-regulation of bi-functional (*katG*) catalase, disarmed the protective response of the organism to oxidative stress generated by the combination of colistin and rafoxanide through ROS, which might be responsible for the significant potentiation of ROS, as well as its enhanced lethality in comparison to colistin treatment alone (7). As antagonism, *UspA* and *UspB*, which have been shown to play an important role in oxidative stress defense (32, 33), have been abundantly expressed in response to antagonizing cell oxidative stress damage and therefore prevent cell lethality caused by the administration.

PMF is associated with the Δ*ψ* gradient and ΔpH, which is reflected in the synthesis of ATP and, in turn, leads to a cascade effect (34, 35). Concretely, since considering the Δ*ψ* did not significantly dissipate after combined administration, the drastic decrease of ATP synthesis would be attributable to other potential mechanisms. Albeit the specific mechanism of ATP synthesis is still unknown, the decrease of ATP concentration eventually invalidated the efflux pumps in lack of sufficient energy. The fluorescence assay of EtBr further demonstrated the depression of efflux pumps. Among Multi-Drug-Resistance (MDR) determinants, Resistance-Nodulation-cell Division (RND) efflux pumps have been reported as the main contributors due to their ability to extrude a wide variety of molecules out of the bacterial cell. *Tol C* and *acr A/B*, the efflux pumps belonging to the RND family, are energy-dependent (36), which, therefore, are directly associated with the intracellular agents’ concentration (i.e., rafoxanide and colistin). Given the overt decrease of ATP concentration in combined administration as we observed in the fluorescence assay, the intracellular agents would therefore aggregate due to the invalidation of efflux pumps and contribute to the bactericidal effect, where the transcriptomic analysis further corroborates our observation in regards to efflux pumps-associated gene expression.

Our study still has several limitations. First, this synergic effect between colistin and rafoxanide is merely proved in *K. pneumoniae* whereas we did not taxonomically choose more objects. Second, only *mgrB*-mediated resistant isolates were included here, and we did not try to make clear the specific synergy mechanism against clinical *mcr*-mediated resistant strains. Further studies should target the synergic mechanism of rescue against *mcr*-mediated strains, and compare the specific mechanism difference against *mgrB*-mediated isolates. Third, as PMF, necessary for ATP synthesis by the F_1_F_0_-ATPase, is not responsible for the ATP synthesis quenching, we failed to find out other proxy factors which may affect ATP.

In summary, we have carried out a series of integrated experiments to verify the specific bactericidal mechanism of the synergic action between colistin and rafoxanide, which involved inner-membrane permeability potentiation, ROS accumulation, and ATP synthesis quenching by inhibiting MDR efflux pumps. The view of transcriptomic responses also corroborated the results of phenotypic screening. Previous research almost used the strains involved in the *mcr*-mediated resistant phenotype, whereas, here, we first investigated the potential synergistic mechanism against nonspecific-colistin-resistant strains hosted in human and swine.

## Data Availability

All the transcriptomic data used in this study have been deposited in the National Center for Biotechnology Information (NCBI) under the accession number PRJNA979835. The complete genomes of KP1 and KP9 were deposited to NCBI’s Sequence Read Archive under the BioProject accession numbers PRJNA994841 (KP1) and PRJNA996717 (KP9).

## SUPPLEMENTAL MATERIAL

Supplemental figures msphere.00234-23-s0001.docx Fig. S1 to S4.

ASM does not own the copyrights to Supplemental Material that may be linked to, or accessed through, an article. The authors have granted ASM a non-exclusive, world-wide license to publish the Supplemental Material files. Please contact the corresponding author directly for reuse.
