# Supplementary material for: The antihelminth drug rafoxanide reverses chromosomal-mediated colistin-resistance in Klebsiella pneumoniae
Source: mSphere. 2023 Sep 25;8(5):e00234-23. doi: 10.1128/msphere.00234-23 (PMC10597454; doi:10.1128/msphere.00234-23)
Supplement: Supplemental figures — Fig. S1 to S4. [file msphere.00234-23-s0001.docx]

**Supplementary materials:**

**Figure S1：**

**
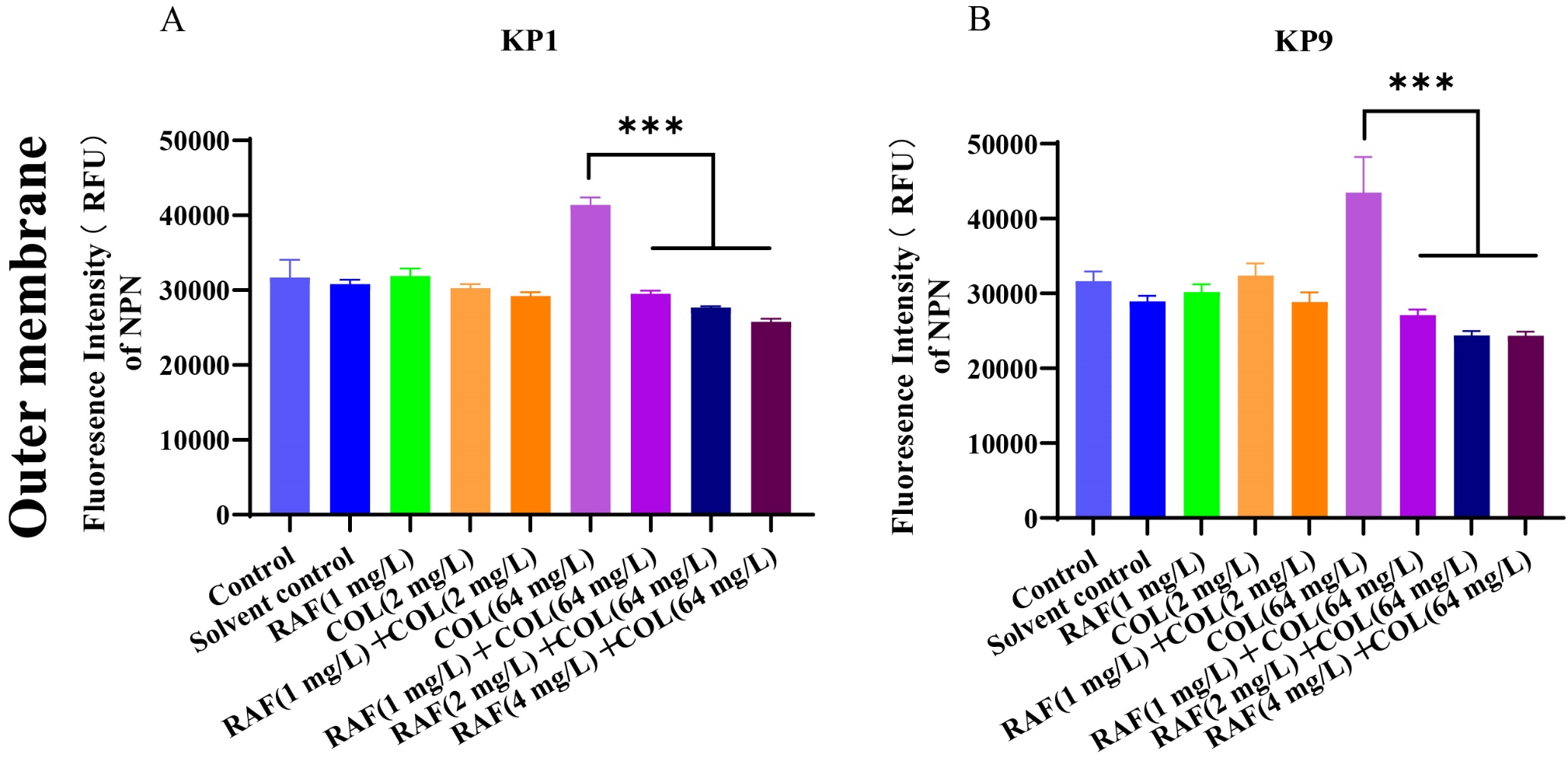
**

**Fig. S1. Extending potential investigation of the interaction between rafoxanide and colistin.** In relation to figure S1A-B, we extended our concentration gradient of rafoxanide to analyze the potential permeability difference of the outer membrane as well as the potential relationship of rafoxanide and colistin in KP1 (A) and KP9 (B). Permeability was evaluated by measuring the fluorescence intensity of propidium iodide after exposure to increasing concentrations of colistin, constant rafoxanide, or colistin plus rafoxanide for 1 h. Unpaired t-test between two groups or one-way ANOVA among multiple groups were used to calculate p-values (*** p < 0.001).

**Figure S2：**

**
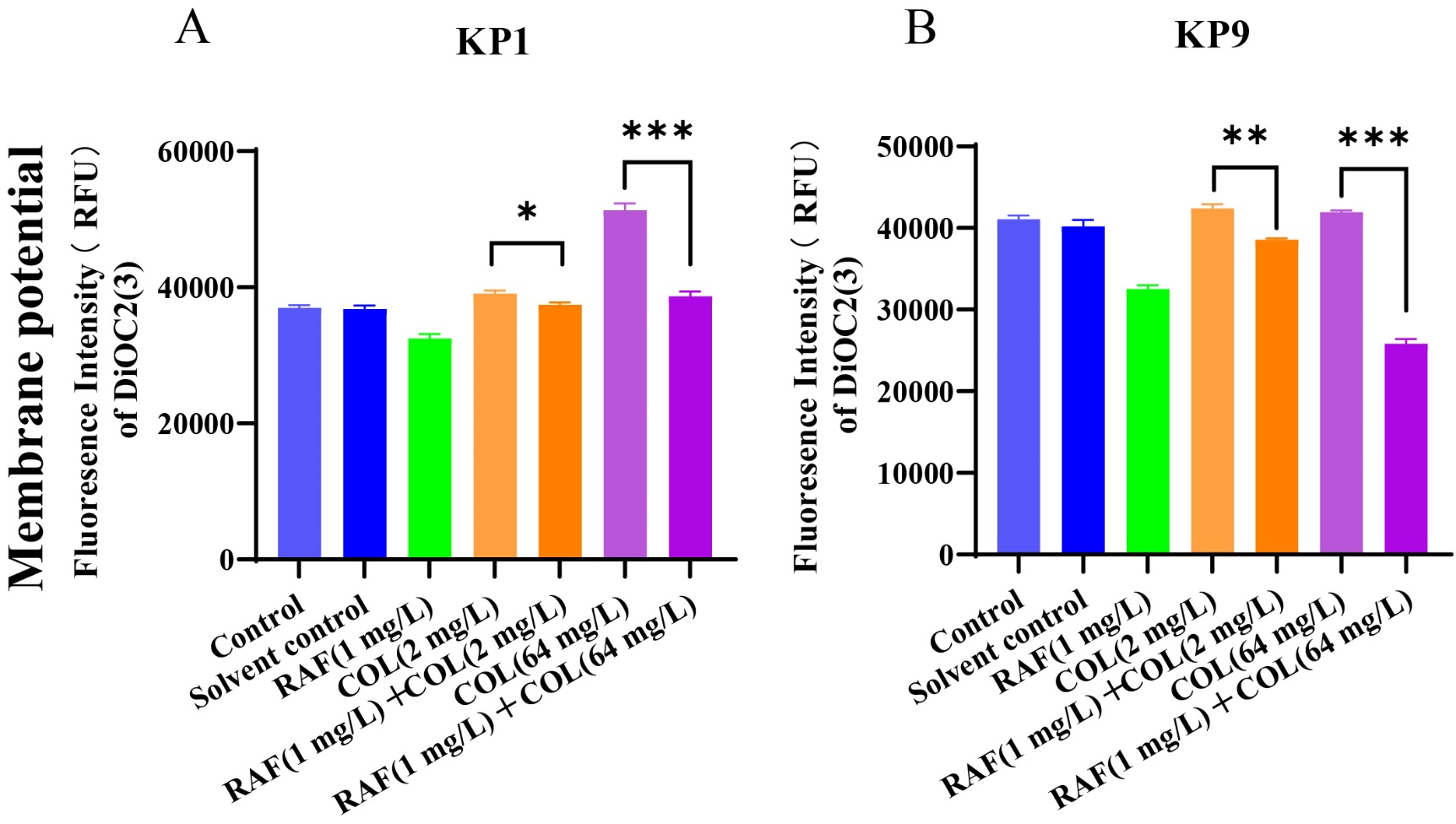
**

**Fig. S2. The membrane potential change treated with rafoxanide and colistin.** Membrane potential changes when KP1 (A) and KP9 (B) were treated with rafoxanide and colistin (1/2 MIC colistin and 1/64 MIC colistin), respectively, or combined administration. Unpaired t-test between two groups or one-way ANOVA among multiple groups were used to calculate p-values (*** p < 0.001).

**Figure S3：**


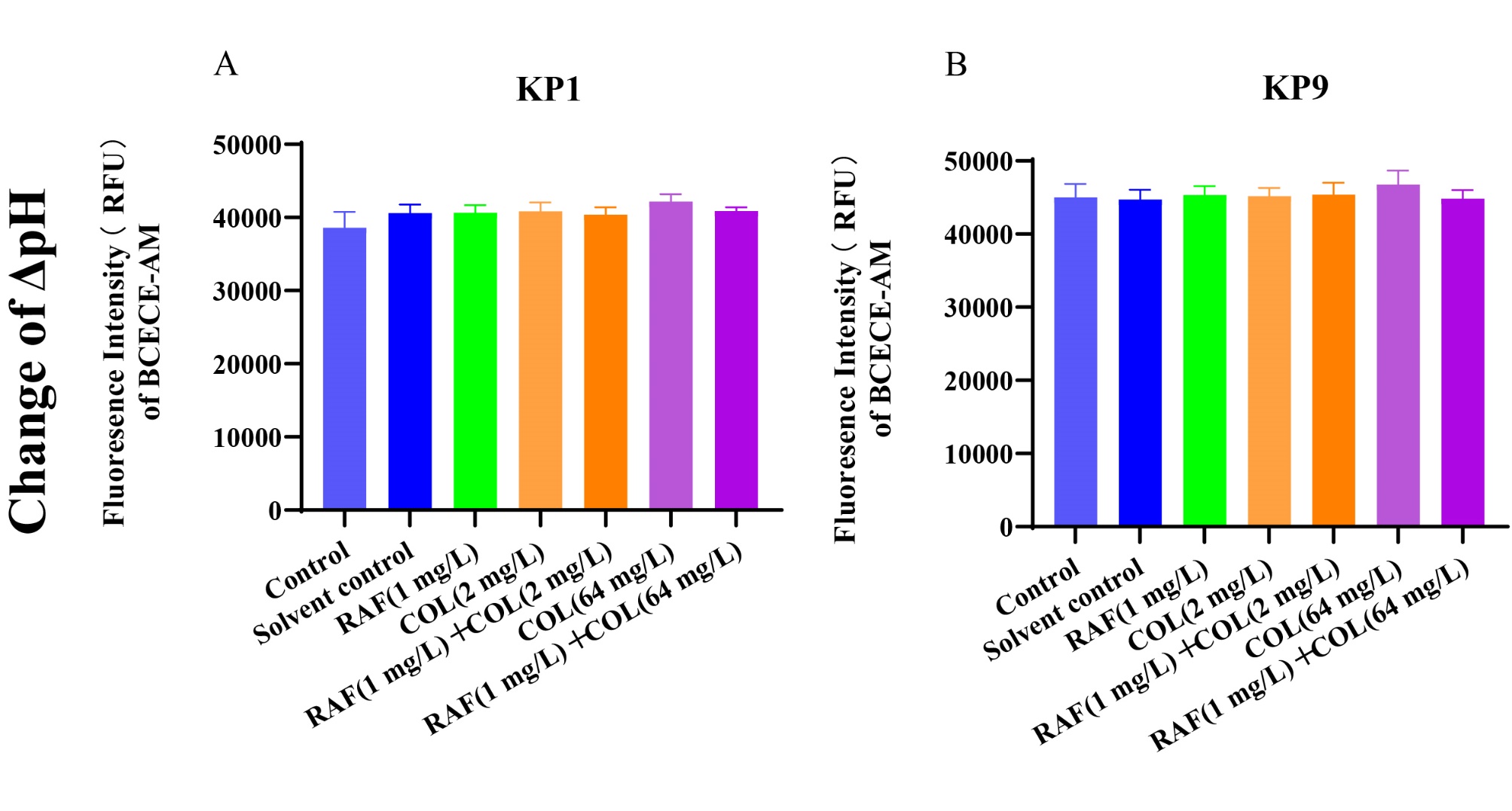


**Fig. S3. The pH change treated with rafoxanide and colistin against KP1 and KP9.** The membrane pH change when KP1 (A) and KP9 (B) were treated with rafoxanide(1 and colistin(1/2 MIC colistin and 1/64 MIC colistin), respectively, or combined administration. Unpaired t-test between two groups or one-way ANOVA among multiple groups were used to calculate p-values (*** p < 0.001).

**Figure S4：**

**
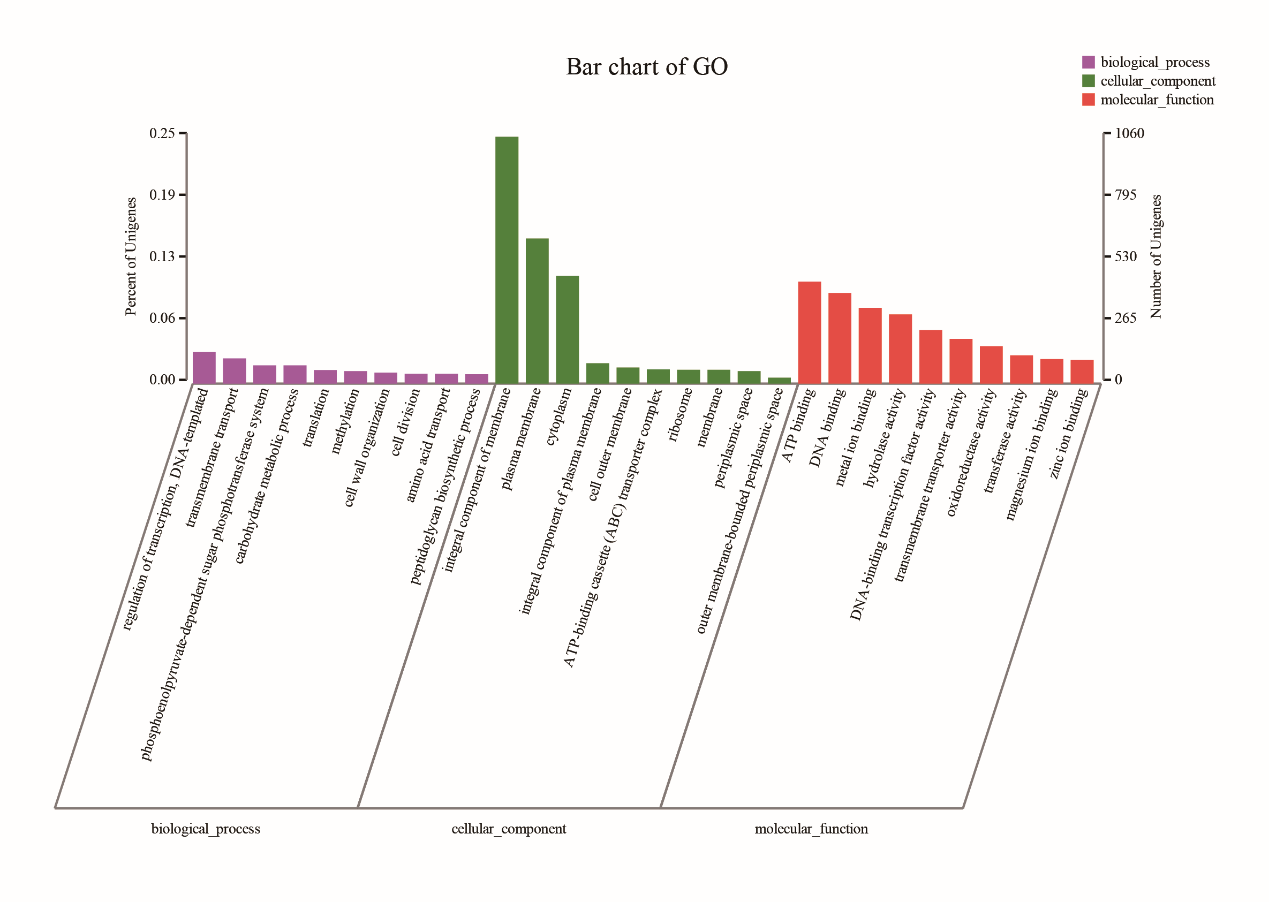
**

**Fig. S4. Functional analysis of transcriptomic profile.** GO functions analysis of differentially expressed genes.
